# Interaction between *COMT* rs5993883 and second generation antipsychotics is linked to decreases in verbal cognition and cognitive control in bipolar disorder

**DOI:** 10.1186/s40359-016-0118-3

**Published:** 2016-04-02

**Authors:** Stephanie A. Flowers, Kelly A. Ryan, Zongshan Lai, Melvin G. McInnis, Vicki L. Ellingrod

**Affiliations:** Clinical Pharmacy Department, College of Pharmacy, University of Michigan, 428 Church St, Ann Arbor, MI 48109-106 USA; Department of Psychiatry, School of Medicine, University of Michigan, 4250 Plymouth Rd, Ann Arbor, MI 48109 USA; Center for Clinical Management Research (CCMR) Veterans Affairs, Ann Arbor, USA

**Keywords:** Cognition, Second generation antipsychotic, *COMT*, Bipolar disorder

## Abstract

**Background:**

Second generation antipsychotics (SGAs) are increasingly utilized in Bipolar Disorder (BD) but are potentially associated with cognitive side effects. Also linked to cognitive deficits associated with SGA-treatment are catechol-O-methyltransferase (*COMT*) gene variants. In this study, we examine the relationship between cognition in SGA use and *COMT* rs5993883 in cohort sample of subjects with BD.

**Methods:**

Interactions between SGA-treatment and *COMT* rs5993883 genotype on cognition was tested using a battery of neuropsychological tests performed in cross-sectional study of 246 bipolar subjects.

**Results:**

The mean age of our sample was 40.15 years and was comprised of 70 % female subjects. Significant demographic differences included gender, hospitalizations, benzodiazepine/antidepressant use and BD-type diagnosis. Linear regressions showed that the *COMT* rs5993883 GG genotype predicted lower verbal learning (*p* = 0.0006) and memory (*p* = 0.0026) scores, and lower scores on a cognitive control task (*p* = 0.004) in SGA-treated subjects. Interestingly, *COMT* GT- or TT-variants showed no intergroup cognitive differences. Further analysis revealed an interaction between SGA-*COMT* GG-genotype for verbal learning (*p* = 0.028), verbal memory (*p* = 0.026) and cognitive control (*p* = 0.0005).

**Conclusions:**

This investigation contributes to previous work demonstrating links between cognition, SGA-treatment and *COMT* rs5993883 in BD subjects. Our analysis shows significant associations between cognitive domains such as verbal-cognition and cognitive control in SGA-treated subjects carrying the *COMT* rs5993883 GG-genotype. Prospective studies are needed to evaluate the clinical significance of these findings.

**Electronic supplementary material:**

The online version of this article (doi:10.1186/s40359-016-0118-3) contains supplementary material, which is available to authorized users.

## Background

Second generation Antipsychotics (SGAs) are distinguished from first generation antipsychotics by the ability to control psychosis at doses associated with considerably fewer extrapyramidal symptoms and a relatively greater 5-HT2A/D2 binding affinity ratio [1]. This class of medication is increasingly utilized in the long-term treatment of Bipolar Disorder (BD) as an alternative monotherapy or more often as an adjunct treatment with lithium or anticonvulsant agents. Significant underlying cognitive deficits in BD patients have not only been observed in manic or depressive episodes but also when euthymic, compared to healthy controls [2–4]. Various medical or lifestyle factors may influence cognitive functioning in this patient population but the contribution of pharmacologic treatment to deficits in cognition remains unclear. In schizophrenia, evidence suggests that cognitive improvements after the initiation of treatment have more to do with practice effects such as exposure, familiarity and/or procedural learning than the implementation of second generation antipsychotics [5]. However, there remains an abundance of recent findings to suggest there are cognitive effects associated with SGA-treatment. Unlike the cognitive benefits observed in some studies for SGA therapy in the schizophrenia population [6–8], evidence indicates that SGAs may have a further detrimental effect on cognition in BD independent of other clinical factors [9, 10]. These data highlight the need to investigate this issue in a large, well-characterized sample of patients with BD.

Previous studies have shown that the regulation of dopamine and dopamine receptors play a role in BD pathophysiology and also in cognitive processes [11, 12]. Contributing to dopamine signaling pathways are both environmental and genetic factors. Catechol-O-methyltransferase (*COMT*) is a major enzyme involved in dopamine metabolism in the prefrontal cortex and has been associated with numerous psychiatric phenotypes [13–15]. The COMT Val108/158Met polymorphism (rs4680) and Val allele load is associated with decreased cognitive performance, such as in executive functioning and working memory in both schizophrenia and BD subjects [16–19]. Although not thoroughly characterized, other *COMT* variants impacting cognition in BD subjects have been described [16, 20]. The objective of this study was to compare neuropsychological performance of SGA vs non-SGA treated bipolar patients with different allelic representation of the *COMT* variants. As previous associations link cognition deficits to treatment with SGAs and *COMT* variant alleles in the BD population, we hypothesize this *COMT* variant would result in decreased cognitive scores in BD patients who are treated with SGAs.

## Methods

### Subjects

The Prechter Longitudinal Study of Bipolar Disorder is an ongoing observational study of bipolar disorder at the University of Michigan (HUM00000606) with the main goal of gathering phenotypic data and biological material [21]. The present study included 246 individuals from this cohort with a DSM-IV diagnosis of BD (BD Type I (*n* = 178), BD Type II (*n* = 39), BD not otherwise specified (NOS, *n* = 21), Schizoaffective disorder-bipolar type (*n* = 8)). All subjects underwent an evaluation using the Diagnostic Interview for Genetic Studies (DIGS; [22]), neuropsychological testing, clinician questionnaires to assess symptoms of depression and mania (Hamilton Depression Rating-17 item (HDRS; [23]) and Young Mania Rating Scale (YMRS; [24]). Diagnoses were confirmed using a best estimate process by at least three MD/PhD clinicians. Medication groups were defined as the use of an SGA at the time of cognitive testing. Second generation antipsychotics, concomitant benzodiazepines and antidepressants used by our cohort are listed in Additional file 1: Table S1. For this cross-sectional analysis, the medication treatment class, neuropsychological performance, age, gender, years of education, time since BD diagnosis, treatment with benzodiazepines or antidepressants and number previous hospitalizations were noted in these subjects.

### Neuropsychological tests

Neuropsychological tests were administered by trained research associates under the supervision of licensed clinicians. The test battery was intended to emphasize known areas affected by BD illness and reported in our prior work [4, 25]. Five specific tests were selected from the original test battery to capture areas that seem to be most sensitive to *COMT* variants. The California Verbal Learning Test-II (CVLT-II, [26]) was used a measure of verbal learning and memory. In this task, five consecutive trials of 16 words are presented and overall learning across the 5 trials was recorded. There was a short-term delayed free recall trial after a distractor list and a long-term delayed free recall trial after 20 min. The Rey-Osterrieth Complex Figure Test (Rey, [27, 28]) was used as a measure of visual learning and memory and required subjects to draw from memory a complex figure that they previously copied and then to recall from memory the figure again after 20 min. To assess executive functioning, the Wisconsin Card Sorting Test (WCST, [29]), a measure of novel problem solving task, and the Trail Making Test (Parts A and B: TMT, [30]), a measure of set-shifting and sequencing, were administered. For the WCST, subjects had to sort cards according to a sorting strategy that they learned based on receiving feedback about prior sorts. Number and type of errors were recorded as well as how many categories sorted. For the TMT Part A, subjects had to manually connect dots in order of numbers that were presented in a spatial array. For the TMT Part B, subjects had to alternate connecting numbers and letters. Total seconds to complete each task was recorded. To assess cognitive control (the ability to engage and disengage in response behaviors), often seen as an element of attention, we used the Parametric Go/No-Go task (PGNG, [31]), a computerized continuous performance test that consists of three separate levels, but only the first level was used for this study. The first level measures attention and response time, resulting in two measures of cognitive control. Subjects respond to a serial stream of letters, pressing a keyboard as quickly as possible whenever they see specific letter.

### Genotyping

Genotyping was done using the HumanCoreExome-12v1 DNA Analysis BeadChip Kit (Illumina, INC., San Diego, CA). Samples were genotyped for greater than 240,000 tagSNP markers and more than 240,000 exome markers by the University of Michigan DNA Sequencing Core. DNAs were quantified using the Quant-iT™ PicoGreen® dsDNA Kit (Invitrogen Corporation, Carlsbad, CA) and samples were assayed according the Illumina Infinium® HD Ultra Protocol. BeadChip image data was recorded using an Illumina iScan Mircroarray Scanner with the Infinium NXT scan setting. Sample image data were analyzed and genotypes determined using the Illumina GenomeStudio (v2011.1) DNA Analysis Software package with Genotyping Modulue (v1.9.4) using the HumanCoreExome-12v1-0_B Manifest and HumanCoreExome-12v1-0_B Cluster file from Illumina. To limit false positives, we conducted a priori analysis with *COMT* due to previous associations with *COMT* variants and cognition. Two SNPS available for analysis were *COMT* rs5993882 and rs165599. Initial data for rs165599 did not show any association with SGA use and cognition and therefore, we focused our analysis on rs5993882.

### Statistical analyses

Hardy–Weinberg equilibrium was tested by a Chi Square analysis. Demographic differences between treatment groups were examined with a standard *t*-test or one-way ANOVA for continuous variables and a chi-square for nominal variables. We performed linear regressions for the multiple variable analyses. For the first linear regression, the cognitive test scores in treatment groups (SGA-treated vs. non-SGA treated) were compared for the three *COMT* rs5993882 genotypes (GG, GT, TT). We adjusted the model for known predictors that may confound cognitive performance, such as age, years of education, gender, diagnosis, benzodiazepine or antidepressant concomitant use, and prior hospitalizations. For tests that were statistically significant, we additionally ran a follow-up analysis using chlorpromazine (CPZ) equivalents as a continuous variable. The second linear regression also was adjusted for these covariates but included new predictors such as *COMT* genotype and an SGA- *COMT* interaction. In the second regression model, the GG *COMT* genotype was used as the comparator for the combined GT and TT genotypes.

Due to the number of cognition test scores, we have adjusted the significance value for regression model 1 using a Bonferroni correction for multiple testing (*p* ≤ 0.0043). For analysis using CPZ-equivalent doses and regression model two, we considered a p value of ≤ 0.05 to be significant. All analyses were conducted in SAS 9.3 (Cary, NC, USA).

## Results

### SNP and haplotype association

Fifty eight patients were homozygous for the *COMT* rs5993882 GG genotype, 120 patients were heterozygous (GT) and 52 patients were homozygous for the TT genotype. No significant deviations from Hardy–Weinberg equilibrium were observed for the *COMT* rs5993882 in the tested population (*p* > 0.5).

#### Study population characteristics

Table 1 represents the demographic parameters of our study population. As cognition can be affected by a number of factors such as age, years of education, gender, diagnosis, severity of BD and concomitant medications, these demographics were used as confounders in our regression models to account for differences between the different genotypes. Our analysis showed significant intergroup differences in gender, concomitant benzodiazepine or antidepressant use, and type of BD diagnosis (see Table 1) with non-SGA treatment group containing more females, less concomitant benzodiazepine and antidepressant use and increased BD-II, BD NOS diagnosed subjects. Mood symptom scores (HAMD, YMRS), recorded at the same time as cognitive testing, showed no statistical differences between treatment populations. As SGAs can be associated with greater severity of BD illness, intergroup variances between time since BD diagnosis and number of previous hospitalizations were also noted. The SGA-treatment group showed a statistically higher number of hospitalizations and this was adjusted for in our regression analysis.

**Table 1:** Demographic characteristics

	NO-SGA^a^	SGA^a^	*p* value
	*N* (%) or	*N* (%) or	
	Mean (SD)	Mean (SD)	
Gender			
Female	119 (69.2)	41(55.4)	0.037
Male	53 (30.8)	33 (44.6)	
Years of education in years (SD)	15.4 (2.9)	15.2 (3.2)	0.63
Age in years (SD)	40.3 (12.9)	40 (11.3)	0.83
Time since diagnosis in years (SD)	14.3 (11.7)	13.9 (10.7)	0.75
Previous hospitalizations	113 (65.7)	58 (78.4)	0.047
Medications			
Benzodiazepines	28 (16.3)	26 (35.1)	0.001
Antidepressants	48 (27.9)	37 (50.0)	0.0008
Chlorpromazine equivalents	NA	210 (787)	
Mood symptoms			
HAMD^b^	8.9 (6.6)	8.2 (6)	0.43
YMRS^c^	3.1 (4.1)	3.2 (3.8)	0.85
Diagnosis			
Bipolar I	115 (66.9)	63 (85.1)	0.014
Bipolar II with recurrent depression	34 (19.7)	5 (6.8)	
Bipolar NOS^d^	18 (10.5)	3 (4.0)	
Schizoaffective, Bipolar	5 (2.9)	3 (4.0)	
*COMT* rs5993883			
GG	44 (25.6)	14 (18.9)	0.5
GT	88 (51.2)	40 (54.1)	
TT	40 (23.2)	20 (27.0)	
Neuropsychological Tests (SD)			
Rey Visual Memory Immediate Recall	21.1 (6.8)	19.1 (6.3)	0.02
Rey Visual Memory Delayed Recall	21.2 (6.5)	19.3 (7.03)	0.043
CVLT-II^e^ Trials 1–5 Score (Learning)	53.9 (11.1)	48.7 (11.4)	0.0009
CVLT-II Short Delay Recall Score	11.4 (3.4)	10.7 (3.7)	0.18
CVLT-II Long Delay Recall Score	12.1 (3.5)	10.5 (3.7)	0.0008
WCST-^f^ Total Errors	23.2 (21.7)	24.5 (22.3)	0.67
WCST- Perseverative Responses (Percentile)	50 (28.9)	49.9 (31)	0.95
WCST-Categories	5.2 (1.7)	5.1 (1.8)	0.53
TMT^g^ A Time (seconds)	29.6 (10.7)	31.2 (11.6)	0.28
TMT B Time (seconds)	71.5 (29.7)	80.8 (34.9)	0.03
PGNG^h^ Response Time (Level 1)	463.5 (50.8)	467.9 (56.5)	0.5
PGNG Target Accuracy (Level 1)	0.9 (0.1)	0.9 (0.1)	0.10

^a^ *SGA* atypical antipsychotic, ^b^ *HAMD* the Hamilton rating scale for depression; ^c^ *YMRS* Young mania rating scale, ^d^ *NOS* not otherwise specified, ^e^ *CVLT-II* California verbal learning test-II, ^f^ *WCST* Wisconsin card sorting test, ^g^ *TMT* trail making test, ^h^ *PGNG* parametric go-no-go test

### Analysis of *COMT* genotypes and cognition in SGA-treated BD patients

We initially examined the association of cognitive deficits in SGA-treated subjects stratified by their *COMT* rs5993883 genotypes (linear regression 1; Table 2). We adjusted this model for age, education, gender, type of BD diagnosis, number of hospitalizations, as well as treatment with benzodiazepines and antidepressants. Our model showed that the GG allele genotype was associated with statistically significant lower scores in specific cognitive domains, such as verbal memory and cognitive control, in subjects treated with an SGA compared to those treated with SGA and with a GT and TT allele. Second generation antipsychotic-treated subjects homozygous for the GG genotype showed a significantly worse CVLT-II verbal learning score when compared to non-SGA treated patients who also carry the GG genotype (*p* = 0.0006; *β* = −10.88; *r*^2^ = 0.51). Although there were no differences between treatment groups for short-term verbal memory (CVLT-II), long-term delayed verbal memory was significantly lower in SGA-treated subjects with the GG genotype (*p* = 0.0026 *β* = −3.43; *r*^2^ = .28) compared to non-SGA treated subjects with the same genotype. The same analysis using CPZ-equivalents found similar findings noting worse CVLT-II verbal learning (*p* = 0.009; *β* = −0.02; *r*^2^ = 0.45) and verbal memory (*p* = 0.016; *β* = −0.009; *r*^2^ = 0.23) in subjects with GG genotypes and higher CPZ-equivalent doses. Subjects treated with SGAs also exhibited lower cognitive control scores as measured by the PGNG-Accuracy score (*p* = 0.004; *β* = 0.083; *r*^2^ = 0.23) compared to those with non-SGA, however, these results were not significant when considering CPZ-equivalents (*p* = 0.1; *β* = −0.0001; *r*^2^ = 0.12) Interestingly, there were no significant cognitive deficiencies between treatment groups when stratified for the heterozygous (GT) or the homozygous minor allele (TT) genotypes.

**Table 2:** Effects of SGA on mean cognitive stores stratified by *COMT* rs5993883 genotype

*COMT* rs5993883	GG (*n* = 58)					GT (*n* = 128)					TT (*n* = 52)				
	No-SGA (STD)	SGA (STD)	beta	*p* value	r^2^	No-SGA (STD)	SGA (STD)	beta	*p* value	r^2^	No-SGA (STD)	SGA (STD)	beta	*p* value	r^2^
Rey Visual Memory Immediate recall	20.9 (6.9)	17.4 (7.2)	−4.13	0.09	0.15	20.7 (6.7)	19.2 (6.4)	−1.8	0.88	0.22	22.2 (6.9)	20.0 (5.5)	−2.2	0.27	0.16
Rey Visual Memory Delayed recall	20.6 (6.9)	17.4 (7.4)	−3.96	0.11	0.14	21.0 (6.2)	19.3 (7.4)	−0.14	0.91	0.19	22.3 (6.8)	20.6 (6.1)	−2.25	0.25	0.23
CVLT-II^a^ learning score	56.6 (10.1)	45.1 (11.7)	10.88	0.0006	0.51	52.0 (12.2)	49.3 (10.9)	−1.77	0.46	0.17	55.2 (8.9)	50.1 (12.0)	−2.77	0.32	0.36
CVLT-II short term delayed free recall	11.6 (3.2)	9.8 (4.5)	−1.6	0.18	0.15	11.0 (3.4)	10.9 (3.5)	−0.12	0.85	0.15	12.0 (3.4)	11.1 (3.8)	−0.68	0.54	0.12
CVLT-II long-term delayed free recall	12.9 (3.1)	9.4 (3.2)	−3.43	0.0026	0.28	11.4 (3.7)	10.7 (3.1)	−0.22	0.75	0.18	12.7 (3.1)	11.0 (3.1)	−0.92	0.29	0.19
WCST^b^ total errors	24.4 (23.5)	27.5 (19.1)	9.3	0.21	0.21	25.1 (22.9)	23.7 (22.9)	−1.17	0.7	0.19	17.7 (15.9)	24.1 (24.2)	6.7	0.23	0.3
WCST perseverative responses	14.3 (15.4)	15.4 (14.6)	4.98	0.33	0.16	15.4 (16.3)	14.1 (16.4)	−1.61	0.62	0.18	9.5 (9.2)	14.5 (17.3)	5.27	0.17	0.25
WCST number of categories	5.2 (1.7)	5.2 (1.3)	−0.4	0.47	0.12	5.1 (1.7)	5.1 (1.9)	0.07	0.83	0.16	5.5 (1.4)	5.0 (1.9)	−0.52	26	0.28
TMT Part A Time (sec)	30.5 (13.3)	34.2 (10.9)	3.73	0.39	0.15	29.6 (9.9)	30.5 (11.9)	0.042	0.98	0.15	28.4 (9.3)	30.5 (11.5)	1.3	0.62	0.4
TMT Part B Time (sec)	75.9 (36.5)	82.1 (30.6)	2.44	0.83	0.16	72.5 (27.4)	80.6 (36.4)	3.5	0.55	0.25	65.2 (25.6)	80.5 (36.5)	10.6	0.2	0.35
PGNG^c^ response time (msec)	468.2 (47.2)	492.2 (61.6)	18.6	0.33	0.19	467.5 (53.4)	463.8 (59.5)	−1.79	0.55	0.2	451.2 (46.8)	459.6 (43.5)	11.3	0.38	0.22
PGNG Target accuracy (%)	0.96 (0.05)	0.88 (0.11)	0.083	0.004	0.23	0.95 (0.07)	0.95 (0.06)	0.002	0.86	0.17	0.98 (0.05)	0.96 (0.07)	−0.02	0.23	0.12

This model was adjusted for age, education, gender, diagnosis, prior hospitalizations, benzodiazepines and antidepressant use

^a^ *CVLT-II* California verbal learning test-II

^b^ *WCST* Wisconsin card sorting test

^c^ *PGNG* parametric go-no-go test

### Interaction of *COMT* rs5993883 genotype GG with SGAs on verbal cognition and impulsivity

Due the observation that these SGA-associated cognitive deficits were only observed in the GG strata, we combined the GT and TT groups and used their scores as a comparator to the GG genotype to measure an interaction between genotype and verbal learning, verbal memory and cognitive control in SGA and non-SGA treatment populations (linear regression 2; Table 3). Also included in this regression model was the contribution of the genotype itself without the SGA-interaction, which combined the GT and TT populations and compared it to the GG genotype. Interestingly, the *COMT* genotype itself was a significant parameter in this model. We also observed a significant interaction between SGA treatment and *COMT* genotype on verbal learning (*p* = 0.028; *β* = 7.95; *r*^2^ = 0.25) and verbal long-term delayed memory (*p* = 0.026 *β* = 2.38; *r*^2^ = 0.21). We also found a significant interaction between genotype and SGA-treatment when examining deficits in cognitive control. (*p* = 0.0005; *β* = 0.083; *r*^2^ = 0.15).

**Table 3:** Interaction between SGA and *COMT* polymorphism rs5993883 on cognition in bipolar patients (using GG genotype as a reference)

Cognitive parameter	Verbal attention^a^ (*r* ^2^ = 0.25)		Verbal delayed recall^a^ (*r* ^2^ = 0.21)		Cognitive control^b^ (*r* ^2^ = 0.15)	
	beta	*p* value	beta	*p* value	beta	*p* value
SGA	−10.03	0.0019	−3.03	0.0022	−0.08	<0.0001
*COMT* genotype	−4.65	0.013	−1.43	0.013	−0.007	0.54
Main Interaction	7.95	0.028	2.38	0.026	0.083	0.0005

This model was adjusted for age, education, gender, diagnosis, prior hospitalizations, benzodiazepines and antidepressant use

^a^Age, education and gender were also significant parameters in this model

^b^Age was also a significant parameter in this model

## Discussion

In this work, we found an association between the GG genotype of *COMT* rs5993883 and SGA-treatment with these individuals with BD showing poorer cognitive performance than those with the GT or TT genotypes. Specifically, we observed significantly lower scores in areas of verbal cognition and cognitive control in this treatment population, indicating that individuals with BD who receive SGA treatment and have the GG genotype are at risk for greater difficulties in learning and remembering verbal or auditory information and they are less accurate when required to engage and disengage their attention to stimuli. Overall, they may be less efficient with learning, memory, and attentional capacity. Although the results of the PGNG Target Accuracy test was not significant when considering CPZ-equivalents, this may be due to non-dose dependent pharmacologic effects. This cohort also exhibited a significant interaction between the SGA-class of medication and *COMT* genotype in the same cognitive domains.

Neuropsychological studies of patients with brain injuries and neuroimaging work has indicated that dopamine action in the prefrontal cortex, dorsal striatum and hippocampus is critical for high level cognitive functioning [32–34]. O-methylation by COMT is one of the major degradative pathways for catecholamine neurotransmitters such as dopamine [15]. Consistent with its role in catecholamine metabolism in the prefrontal cortex, variation in this gene has been linked with decreased cognitive function in BD, schizophrenia and in healthy controls [14, 16, 35]. The most widely studied *COMT* variant allele is the COMT Val108/158Met polymorphism rs4680. This variant affects the stability and enzymatic activity of catechol-O-methyltransferase, which alters the enzyme's ability to methylate catecholamines in the pre-frontal cortex [36–38]. In previous work, Val allele load has been associated with detrimental effects in cognition for schizophrenia subjects and has also been linked to a further decrease in cognition in BD patients treated with SGAs [9].

The polymorphism *COMT* rs5993883 is located in intron 1 of the *COMT* gene and is not strongly linked to the rs4680 polymorphism (Distance = 13633 base pairs; *r*^2^ = .327; d’ = 0.654; www.broadinstitute.org/mpg/snap/). In previous work, this mutation has been weakly associated with creativity, cocaine induced paranoia and modulation of certain personality traits including suicidal behavior [15, 39, 40]. Additionally, the rs5993883 G allele has been associated with cognitive manic symptoms in BD patients [41]. Intron variants are not in the protein-coding region of a gene but can generally affect function by altering processes such as transcription or alternative splicing, in which several splice variants have been noted for COMT [42–44]. Although no structural or transcriptional changes in function have been defined for *COMT* rs5993883, it’s possible that this variant could affect these types of processes.

Impairments in cognition are noted as being robustly evident in the schizophrenia literature but have also been noted in BD patients, although to a lesser degree. When compared to healthy controls, euthymic BD patients show deficiency in executive functioning, verbal memory, psychomotor speed and sustained attention [45]. It has also been observed that in first degree relatives, the cognitive domains of executive functioning and verbal memory are significantly different from healthy controls, which suggests these domains are bipolar endophenotypes reflecting a genetic link to BD [46]. Impaired inhibitory behavioral control in manic and euthymic BD subjects is a specific cognitive impairment that has also been described as distinct from the universal neuropsychological deficiency linked to other psychotic disorders [47, 48]. The overall cause of neurocognitive deficits in BD patients is likely multifactorial including genetic, medication and symptom considerations. Although deficiencies in verbal memory have been described in the BD population, we have observed further decrements in this domain due to an interaction between SGA-treatment and the GG genotype of the *COMT* rs5993883 variant. We also described a relationship between *COMT* rs5993883 and SGA-treatment on deficiencies in cognitive control as measured by a continuous performance test (PGNG).

Second generation antipsychotics have a role in the management of not only BD-associated mania but are also effective in BP-associated depression. Although the mechanistic basis for the efficacy of SGAs in mood disorders is not completely understood, the ability to block D2 and serotonin 5HT_2A_ receptors are likely to contribute. Dopamine dysregulation is thought have a role in the psychopathology of BD [49]. However, in contrast to the cognitive improvement observed in SGA-treated schizophrenia patients, SGAs use within the BD population has been associated with lower cognitive functioning. As a further complication for cognition in this group, our work and others have shown that treatment with SGAs may confer further decrements in cognition if the subject caries *COMT* variants [9]. In this report, we observe that a well-characterized large group of BD subjects show significantly lower cognitive performance in specific domains of verbal cognition and cognitive control that are associated with an SGA-treatment interaction with a GG genotype of *COMT* rs5993883.

### Study limitations

As this study was cross-sectional in design, we miss looking longitudinally at cognitive measures in APP-treated subjects over time. In the future, as we accumulate more data in the Prechter longitudinal cohort, a longitudinal analytic approach will be informative. Additionally, we know that members of the SGA-class are not identical in either the mechanism of action or side effects. In this study, we did not distinguish between specific SGA-medications but this may be warranted in future work.

Greater severity of illness is associated with SGA treatment in the BD population, which can also result in reduced cognitive functioning. In an attempt to address this disparity, we adjusted our model for prior hospitalizations, as an indicator of disease severity, which showed a statistically significant increase in the SGA-treated population. However, it may also be important to consider other factors such as medication switching or chlorpromazine equivalents for SGA use to assess severity of illness. And finally, we also had a significantly under representation of the BD-II and schizoaffective BD type diagnoses when compared to subjects with a BD-I diagnosis. There may be differences in the effect of *COMT* variant alleles and interactions with SGA-treatment in the less-represented diagnosis in our subject cohort. As we accrue more subjects, this analysis may be possible using the Prechter cohort.

## Conclusions

This investigation contributes to work illustrating links between cognition, SGA-treatment and *COMT* in BD subjects. Our analysis highlights significant associations between decreased verbal-cognition and cognitive control in SGA-treated subjects carrying the *COMT* rs5993883 GG-genotype. Prospective studies are needed to assess the clinical importance of these findings.

### Ethics approval and consent to participate

The Prechter Lonigtudinal Study of Bipolar Disorder has been approved by the University of Michigan Institutional Review Board (HUM00000606).

### Availability of data and materials

The the Heinz C. Prechter Bipolar Research study is an ongoing study. Materials are not public at this time.

## Additional file

Additional file 1: Table S1. Atypical Antipsychotics, benzodiazepines and antidepressants used in this study. (XLS 9 kb)

## Abbreviations

BD: bipolar disorder
*COMT*: catechol-o-methyltransferase
CVLT-II: The California verbal learning test-II
DIGS: diagnostic interview for genetic studies
HDRS: Hamilton depression rating-17 item
NOS: not otherwise specified
PGNG: parametric go/no-go task
Rey: The Rey-Osterrieth complex figure test
SGAs: second generation antipsychotics
TMT: trail making test parts A and B
WCST: Wisconsin card sorting test
YMRS: Young mania rating scale
